# Evaluation of the Inflammatory Cytokines and IL-10 Network in Individuals Co-infected With Human T-Cell Lymphotropic Virus and Hepatitis C Virus (HTLV/HCV)

**DOI:** 10.3389/fmicb.2021.632695

**Published:** 2021-02-26

**Authors:** Felicidade Mota Pereira, Pablo Ivan Pereira Ramos, Monique Lirio, Ajax Mercês Atta, Isabela Silva de Oliveira, Fabio Carneiro Vosqui Nascimento, Marcelo Costa Silva, Bernardo Galvão-Castro, Maria Fernanda Rios Grassi

**Affiliations:** ^1^Laboratório Avançado de Saúde Pública, Fundação Oswaldo Cruz, Salvador, Brazil; ^2^Laboratório Central de Saúde Pública Prof. Gonçalo Moniz – Secretaria da Saúde do Estado da Bahia, Salvador, Brazil; ^3^Centro de Integração de Dados e Conhecimentos para Saúde – CIDACS, Fundação Oswaldo Cruz, Salvador, Brazil; ^4^Faculdade de Farmácia, Universidade Federal da Bahia, Salvador, Brazil; ^5^Secretaria Municipal de Saúde de Feira de Santana, Feira de Santana, Brazil; ^6^Secretaria da Saúde do Estado da Bahia, Salvador, Brazil; ^7^Escola Bahiana de Medicina e Saúde Pública, Salvador, Brazil

**Keywords:** HTLV, HCV, co-infection, network, cytokines

## Abstract

**Background:**

Co-infection between the human T-cell lymphotropic virus (HTLV) and the hepatitis C virus (HCV) can modify the natural history of HCV infection. The aim of this study was to describe the inflammatory cytokines and IL-10 network in patients co-infected with HTLV and HCV viruses in Bahia, Brazil.

**Methods:**

Samples from 31 HTLV/HCV co-infected individuals and 27 HCV monoinfected individuals were evaluated. IFN-γ, TNF-α, IL-10, IL-8, and IL-1 cytokines were quantified by ELISA. Clinical, laboratory data were obtained from patient records. Serum levels of the cytokines were log_10_-transformed and data mining was performed using Z-score statistics and correlation analysis.

**Results:**

Co-infected individuals presented a tendency toward higher production of INF-γ compared to the HCV monoinfected group. Regarding cytokine pairs, there was a positive correlation (*P*-value < 0.05) between IL-1 and IL-8 in the HTLV/HCV co-infected group and uninfected controls, and two correlations in the HCV mono-infected group IL-8 – IL10 and IL- INF-γ – IL-10 pairs. There was no significant difference between the groups for the other parameters analyzed.

**Conclusion:**

The results presented herein indicated that HTLV/HCV co-infection was associated with a trend in IFN-γ production while HCV-infected individuals presented a positive correlation with both inflammatory cytokines (IL-8 and IFN-γ) and the regulatory cytokine IL-10.

## Introduction

Co-infection with Human T-Cell Leukemia Virus type 1 (HTLV-1) or type 2 (HTLV-2) and the hepatitis C virus (HCV) has been described in countries where both viruses are endemic (Nakashima et al., 1994), occurring mainly among drug users (de la Fuente et al., 2006; Zunt et al., 2006; Berini et al., 2007; Moreira et al., 2013). In Brazil, the prevalence of HTLV-1 varies according to the geographic region, in Bahia it is estimated that around 1% (∼130,000) people are infected by this virus (Pereira et al., 2019). The prevalence of HTLV infection reaches 5.3% in HCV carriers (Caterino-de-Araujo et al., 2018) and HTLV/HCV co-infection was identified in 1.5% in men who have sex with men (Catalan-Soares et al., 2014).

The clinical progression and immunological features of HTLV/HCV co-infection remain poorly understood and vary in accordance with geographic region. Some studies have shown that HTLV-1/HCV co-infection has a synergistic effect on liver disease severity and lethality (Kishihara et al., 2001). In Japan, several reports suggest that the presence of HTLV-1 negatively influences the natural course of HCV infection by reducing the cellular immune response against HCV and decreasing the cytotoxic action of T-lymphocytes against infected hepatocytes (Boschi-Pinto et al., 2000). In addition, it was reported that HTLV/HCV co-infection worsened the progression of HCV infection to hepatocellular carcinoma (Kamihira et al., 1991; Tokunaga et al., 2014), and decreased the sustained virological response of interferon treatment against hepatitis C (Kishihara et al., 2001). In contrast, studies conducted in Brazil have shown that HTLV may contribute to the spontaneous clearance of HCV in co-infected individuals (Moreira et al., 2013; Le Marchand et al., 2015). Some co-infected individuals evaluated in Brazil present less hepatic fibrosis, higher CD4^+^ T-cell counts and lower alanine aminotransferase levels compared with HCV-infected individuals alone (Bahia et al., 2011; Abad-Fernandez et al., 2015; Espindola et al., 2017).

While few studies have investigated cytokine profiles in the context of HTLV/HCV co-infection, increased serum levels of IFN-γ were reported in HTLV/HCV co-infected patients compared to those with HCV alone (Silva et al., 2016). Moreover, individuals with triple HTLV/HIV/HCV infection present increased plasma levels of proinflammatory cytokines compared to others with HIV-HCV co-infection (Brites et al., 2018). The present study aimed to describe inflammatory and regulatory cytokine profiles in a group of HTLV/HCV co-infected patients, and compare the obtained results with individuals infected with HCV alone.

## Materials and Methods

### Ethics Statement

The Institutional Review Board (IRB) for Human Research at the Gonçalo Moniz Institute of the Oswaldo Cruz Foundation (Salvador, Bahia, Brazil) provided ethical approval to conduct this study (CAAE number 22478813.7.0000.0040).

### Study Population

The study was conducted at the Central Laboratory of Public Health of the state of Bahia (LACEN/BA), from January 2014 to June 2016. All samples of individuals tested for HCV viral load quantification were sequentially selected. Individuals were included if they had been previously tested for HTLV serology (confirmed by Western blot) at LACEN. Subjects were distributed into two groups: Samples from 31 HTLV/HCV co-infected individuals and 27 HCV monoinfected individuals were evaluated. Samples with a reagent result for HIV were excluded.

### Data Collection

Socio demographic data and serological results for HTLV and HIV were obtained from the SMART LAB database that is the laboratory management system of LACEN-BA. Clinical information on inflammatory liver activity and degree of fibrosis [both evaluated by the METAVIR scale (Bedossa and Poynard, 1996)], presence of hepatic steatosis, ascites, hepatic encephalopathy, antiretroviral treatment (ART) to hepatitis C, platelet count and prothrombin time were obtained from the patients’ medical records. No clinical status of HTLV patients was available.

### HCV Viral Load and Genotyping

Plasma samples were collected in tubes containing EDTAK3. HCV viral load was quantified using the Abbott RealTime HCV kit (ABBOTT Molecular Inc., Des Plaines, United States) and the determination of viral genotype was performed using the Abbott RealTime HCV Genotype II (ABBOTT Molecular Inc., Des Plaines, United States).

### Detection of Cytokines

Cytokines were measured in the plasma of HCV- and HTLV/HCV-infected individuals. A control group consisting of 30 healthy individuals with non-reactive results for HTLV, HCV, HIV, and demographic characteristics similar to those in the HCV and HTLV/HCV groups was selected from the sample bank of the Immunology Laboratory of the Pharmacy School of the Federal University of Bahia. The levels of INF-γ, TNF, and IL-10, were measured using the Enzyme-linked Immunosorbent Assay kits (eBioscience, Bender, Vienna, Austria). The levels of IL-1 and IL-8 were measured using the Uncoated ELISA kits (Invitrogen, Bender, Vienna, Austria) in accordance with the manufacturer’s instructions.

### Data Analysis

Prism^®^ Version 5.03 (GraphPad) was used for data analysis. The categorical variables (gender, inflammatory activity, degree of fibrosis, treatment, presence of steatosis, hepatic encephalopathy, as well as ascites) were presented as absolute and relative frequencies. The continuous variables (age, HCV viral load, platelet count, and prothrombin time) as central tendency and dispersion measures (mean, standard deviation, median and interquartile range, confidence interval). To compare proportions among categorical variables, the chi-square test (χ^2^), ANOVA or Fisher’s exact test were used. To compare continuous variables, the *t*-test for independent samples was used. The analyzes were bilateral (two-tailed), and a value of *P* ≤ 0.05 was considered statistically significant.

### Data Mining of Cytokine Profiles

Serum levels of the cytokines IFN-γ, TNF-α, IL-10, IL-8, and IL-1 of the evaluated groups were log_10_-transformed prior to data mining using Z-score statistics and correlation analysis. In order to avoid log evaluation errors, the values equal to zero were replaced by the detection limit of the instrument according to the manufacturer. Conversely, the values exceeding the upper limit were replaced by the upper limit of detection. Deviations from the mean were calculated as Z-scores aggregated across groups using the *scale* function in R 3.2.2 (R Core Team, 2018), allowing to disclose trend changes in cytokine profiles. Additionally, correlation analysis was performed to infer within-group associations between cytokine-cytokine pairs.

The R package *qpgraph* v. 2.10.2 (Castelo and Roverato, 2006) was used to calculate Pearson’s correlation statistic and corresponding *P*-values.

In order to disclose a network from these associations, the package *igraph* v. 1.2.2 (Csardi and Nepusz, 2006) was used based on a nominal correlation *P*-value threshold of <0.05 to establish an edge between cytokine nodes, with self-loops removed for clarity.

## Results

A total of 58 individuals (31 HTLV/HCV and 27 HCV) and 30 uninfected controls were included. No statistically significant differences were seen between individuals with HTLV/HCV co-infection, HCV-infected patients and controls with respect median age and sex. Most co-infected individuals resided in the municipality of Salvador (*P* < 0.0001) (Table 1).

**TABLE 1:** Demographic characteristics of HTLV/HCV-, HCV-infected patients and controls.

Variable	HTLV/HCV *N* = 31	HCV *N* = 27	CONTROL *N* = 30	*P*-value
Age (years)	61 (55–68)	63 (56–69)	61 (56–67)	0.6598
**Sex, *n* (%)**				
Male	17 (55)	14 (52)	15 (50)	0.9297
Female	14 (45)	13 (48)	15 (50)	
**City of origin, N (%)**				<0.0001
Salvador	21 (68)	11 (41)	30 (100)	
Other	10 (32)	16 (59)	0	

*Data presented as number (percentage), except age (median and interquartile range).*

Regarding clinical data, the frequencies of steatosis, ascites and neurological findings suggestive of hepatic encephalopathy were similar between the HTLV/HCV and HCV groups. No statistically significant differences were found between these groups in the proportion of non-responders to HCV ART, yet this proportion was higher in HCV-infected individuals (44%) compared to HTLV/HCV (35%) (*P* = 0.72) (Table 2). Liver biopsy results were available from 21 out of 31 HTLV/HCV co-infected individuals and 14 out of 27 HCV-infected individuals. Moderate/severe inflammatory activity (A2-A3) was observed in 35% (7/20) of the HTLV/HCV individuals and 43% (6/14) of HCV patients. The frequency of advanced fibrosis (F3-F4) was slightly lower in the HTLV/HCV (43%, 9/21) group compared to HCV-infected (64%, 9/14) individuals. Liver cirrhosis was identified in two individuals in each group.

**TABLE 2:** Clinical and histopathological findings of HTLV/HCV-co-infected and HCV-infected individuals.

Variable	HTLV/HCV		HCV		*P*-value
	N	*n* (%)	N	*n* (%)	
HCV ART (non-responders)	17	6 (35)	16	7 (44)	0.72
Liver inflammatory activity (A2-A3)*	20	7 (35)	14	6 (43)	0.73
Liver fibrosis (F3-F4)*	21	9 (43)	14	9 (64)	0.31

*METAVIR scale; comparisons made using Fisher’s exact test.

**FIGURE 1:**
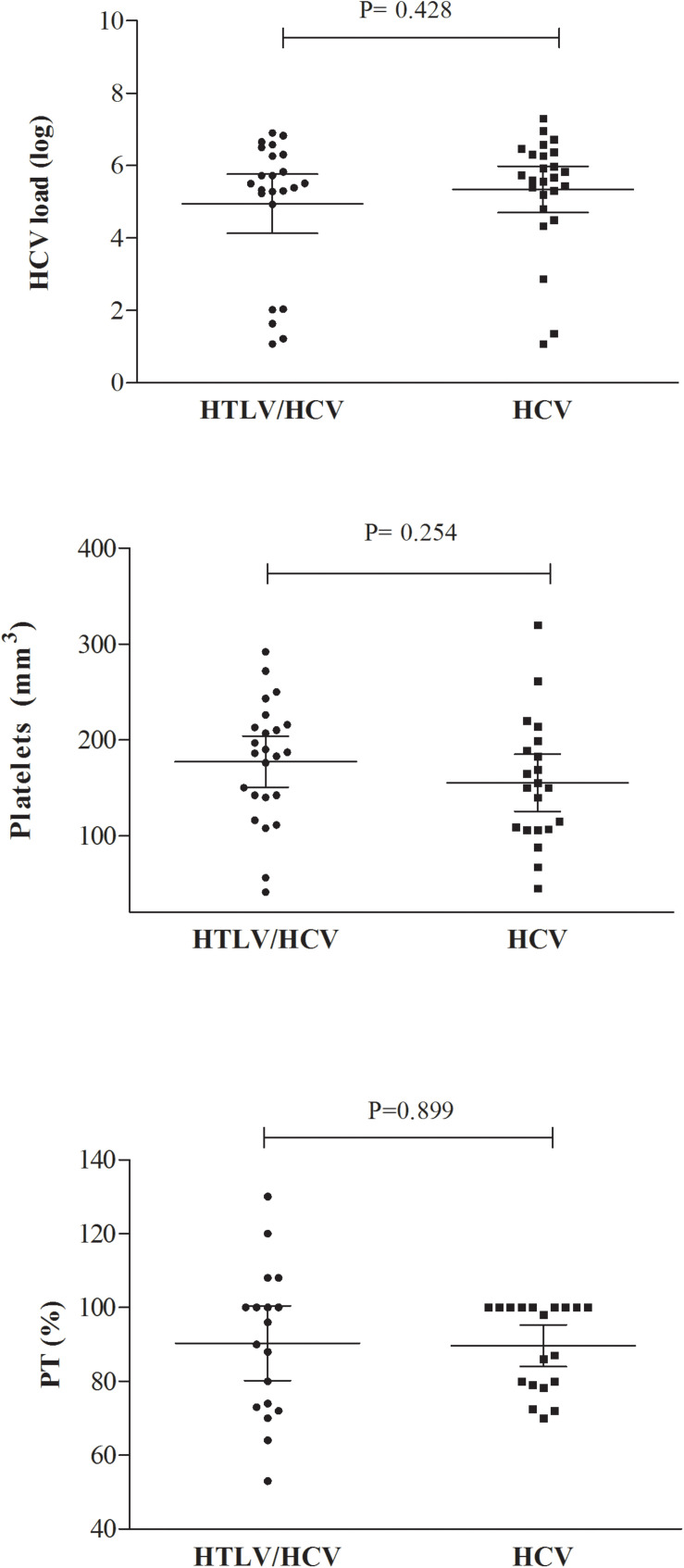
HCV load, platelet count, and prothrombin time (PT) in HTLV/HCV-co-infected and HCV-infected groups.

No significant differences were observed in mean HCV viral load between the HTLV/HCV (5.3 ± 1.9 log) and HCV (4.9 ± 1.5 log) groups (*P* = 0.428). Viral load was more frequently undetectable in the HTLV/HCV group (26%, 8/31) compared to HCV (7.4%, 2/27) (*P* = 0.08). Mean platelet count and prothrombin time were similar between the groups (Figure 1).

Hepatitis C virus genotype 1 was identified in 74.2% (23/31) and 85.2% (23/27) of the HTLV/HCV and HCV groups, respectively, while genotype 2 was found in 3.2% (1/31) of the HTLV/HCV group. Genotype 3 was detected in 9.7% (3/31) and 7.4% (2/27) of individuals with HTLV/HCV and HCV infection, respectively.

**FIGURE 2:**
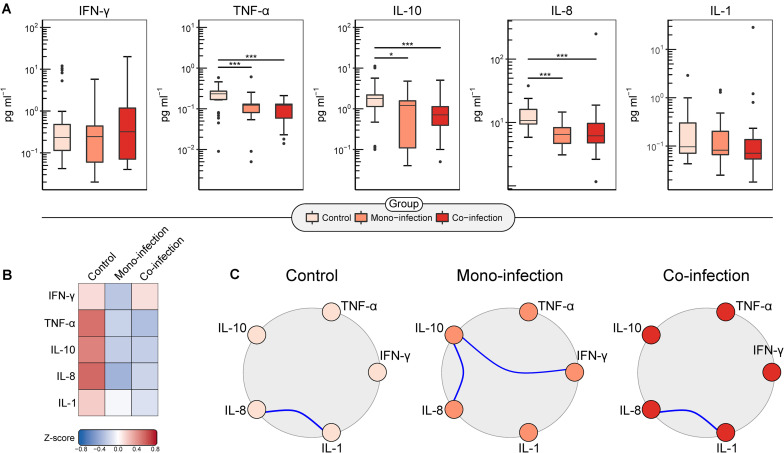
Analysis of cytokine expression among the compared groups. **(A)** Boxplots of serum levels (pg/mL) of log10-transformed IFN-γ, TNF, IL-10, IL-8, and IL-1 cytokines. *p < 0.05, ***p < 0.001 (Kruskal–Wallis with Dunn’s post-test and Holm’s correction for *P*-value adjustment). **(B)** Heatmap generated from *Z*-scores calculated across groups illustrates differences in cytokine profiles among the compared groups. **(C)** Cytokine networks constructed using Pearson’s correlation coefficient depict pairwise relationships between cytokine expression levels. Blue-colored links between nodes represent statistically significant positive correlations.

With respect to cytokine levels, both HTLV/HCV and HCV groups presented lower levels of TNF, IL-10, and IL-8 compared to uninfected controls (*P*-value < 0.001) (Figure 2A). Then, a Z-score analysis was performed to identify subtler deviations in cytokine levels across all groups. A trend toward higher IFN-γ production was observed in the HTLV/HCV group compared to HCV (Figure 2B), albeit this difference was not supported by statistical significance. With respect to cytokine pairs (Figure 2C), statistically significant associations were identified between IL-1 and IL-8 in the uninfected control group and the HTLV/HCV co-infected group. Two statistically significant associations between IL-8/IL-10 and IFN-γ/IL-10 were found in the HCV group (Figure 2).

## Discussion

The present study aimed to evaluate aspects of HTLV/HCV co-infection in comparison to HCV infection alone. We evaluated inflammatory (INF-γ, TNF, IL-1, and IL-8) and regulatory (IL-10) cytokine networks in HTLV/HCV-co-infected and HCV-infected individuals. Cytokine production was found to be similar between these groups, yet TNF, IL-8, and TNF levels were lower than in uninfected controls. Similar results have been described in other studies reporting on HCV infection, as HTLV co-infection was not found to influence cytokine levels (Abad-Fernandez et al., 2015; Silva et al., 2016). However, the present evaluation of trend changes in cytokine profile between infected and co-infected groups identified higher IFN-γ production in the HTLV/HCV group compared to HCV. Indeed, HTLV-1 infection has been strongly associated with higher inflammatory cytokine production, especially INF-γ (Carvalho et al., 2001), which could explain the relatively increased production of this cytokine in HTLV/HCV-infected individuals.

The evaluation of cytokine networks revealed a similar profile between HTLV/HCV-co-infected individuals and uninfected controls, with a positive association identified between IL-1 and IL-8. By contrast, a positive correlation between the IL-8/IL-10 and INF-γ/IL-10 pairs was identified in the HCV-infected group. IL-8 levels have been reported to be inversely correlated with liver disease activity, since reduced concentrations of this cytokine are observed following effective antiviral treatment (Alhetheel et al., 2016). IFN-γ is directly associated with an effective cytotoxic response to HCV and to the control of virus replication (Thimme et al., 2006). On the other hand, high levels of IL-10 were shown to lead to an impairment in cytotoxic response, decreased inflammatory response and progression to liver fibrosis (Nelson et al., 2000; Mege et al., 2006; Aroucha et al., 2013; de Souza-Cruz et al., 2016).

The suppressor effect of IL-10 could be responsible for the lower cytotoxic response and higher HCV viral loads seen inpatients infected with HCV alone. In fact, despite a lack of statistical significance, 26% of HTLV/HCV-co-infected individuals had undetectable viral load versus just 7.4% in the HCV group (*P* = 0.08). Moreover, more HCV-infected patients were non-responders to the treatment, with high inflammatory liver activity and advanced liver fibrosis. In Brazil, some studies have suggested that HTLV co-infection may have a positive effect on spontaneous HCV clearance (Alves et al., 2018), which occurs equally in patients triple infected with HTLV, HCV, and HIV-1 (Bahia et al., 2011; Moreira et al., 2013; Le Marchand et al., 2015). However, a study conducted in Japan reported that HTLV decreased the response to the treatment with IFN-α and reduced the clearance of HCV (Kishihara et al., 2001). These conflicting results may be due to differences in the therapeutic protocols used in Japan and Brazil that combines antivirals with the IFN-α.

In addition, differences in the viral genotype may also influence the response to the treatment. In the present study, HCV genotype 1 was the most frequent in both HCV and HTLV/HCV co-infected groups. Similar results were reported in other studies conducted in Salvador, Bahia, and Rio de Janeiro (Silva et al., 2016; Espindola et al., 2017). This genotype is commonly associated with failure to antiviral treatment (Simmonds et al., 1996, 2005). However, most patients in the present study were responsive to treatment and had few advanced fibrosis and high inflammatory activity.

The present study is limited by its study design that does not allow to make any causal and temporal association. The majority of included patients were under ART for HCV infection and had a partial control of the viremia. It is possible that the cytokine network between inflammatory/regulatory cytokines of naïve patients was modified by therapy. In addition, a HTLV single-infected patients group for comparison purposes was not included, and the impact of HCV on HTLV infection could not be assessed.

## Conclusion

In conclusion, the results presented herein indicated that HTLV/HCV co-infection was associated with a trend to IFN-γ production while HCV-infected individuals presented a positive correlation between both inflammatory cytokines (IL-8 and IFN-γ) and the regulatory cytokine IL-10. The trend of higher IFN-γ levels observed in HTLV/HCV co-infected individuals may be related to the partial control of the viremia in these individuals, with a greater proportion of undetectable viral load and clinical responders to the treatment.

## Data Availability Statement

The raw data supporting the conclusions of this article will be made available by the authors, without undue reservation.

## Ethics Statement

The studies involving human participants were reviewed and approved by the Institutional Research Board of Fiocruz. Written informed consent for participation was not required for this study in accordance with the national legislation and the institutional requirements.

## Author Contributions

FP, PR, AM, BG-C, and MG: study conception and design. FP, PR, ML, IS, FN, MS, and MG: acquisition of data. FP, PR, ML, AM, IS, FN, MS, BG-C, and MG: analysis and interpretation of data. FP, PR, BG-C, and MG: drafting of the manuscript and critical revision. All authors have read and approved the final version of the manuscript.

## Conflict of Interest

The authors declare that the research was conducted in the absence of any commercial or financial relationships that could be construed as a potential conflict of interest.
